# Substantial impact of 3-iodothyronamine (T1AM) on the regulations of fluorescent thermoprobe-measured cellular temperature and natriuretic peptide expression in cardiomyocytes

**DOI:** 10.1038/s41598-022-17086-2

**Published:** 2022-07-26

**Authors:** Hirotake Takahashi, Tomohisa Nagoshi, Haruka Kimura, Yoshiro Tanaka, Rei Yasutake, Yuhei Oi, Akira Yoshii, Toshikazu D. Tanaka, Yusuke Kashiwagi, Michihiro Yoshimura

**Affiliations:** Division of Cardiology, Department of Internal Medicine, The Jikei University School of Medicine, 3-25-8, Nishi-Shimbashi, Minato-ku, Tokyo, 105-8461 Japan

**Keywords:** Heart failure, Preclinical research, Endocrine system and metabolic diseases

## Abstract

There is growing interest in 3-iodothyronamine (T1AM), an active thyroid hormone metabolite, that induces negative inotropic and chronotropic actions in the heart and exerts systemic hypothermic action. We explored the direct impact of T1AM on cardiomyocytes with a focus on the regulation of the intracellular temperature and natriuretic peptide (NP) expression. A thermoprobe was successfully introduced into neonatal rat cardiomyocytes, and the temperature-dependent changes in the fluorescence intensity ratio were measured using a fluorescence microscope. After one-hour incubation with T1AM, the degree of change in the fluorescence intensity ratio was significantly lower in T1AM-treated cardiomyocytes than in equivalent solvent-treated controls (*P* < 0.01), indicating the direct hypothermic action of T1AM on cardiomyocytes. Furthermore, T1AM treatment upregulated B-type NP (BNP) gene expression comparable to treatment with endothelin-1 or phenylephrine. Of note, ERK phosphorylation was markedly increased after T1AM treatment, and inhibition of ERK phosphorylation by an MEK inhibitor completely cancelled both T1AM-induced decrease in thermoprobe-measured temperature and the increase in BNP expression. In summary, T1AM decreases fluorescent thermoprobe-measured temperatures (estimated intracellular temperatures) and increases BNP expression in cardiomyocytes by activating the MEK/ERK pathway. The present findings provide new insight into the direct myocardial cellular actions of T1AM in patients with severe heart failure.

## Introduction

Although thyroid hormones (triiodothyronine [T3] and its precursor thyroxine [T4]) have a well-established role in myocardial contractility and hemodynamics, increasing attention is being paid to active thyroid hormone metabolites, particularly 3-iodothyronamine (T1AM)^1–4^. T1AM is a decarboxylated and deiodinated thyroid hormone derivative, although a biosynthetic pathway for T1AM has not yet been clearly identified^2^. Intriguingly, a cross-sectional study demonstrated that serum T1AM levels were increased in heart failure subjects with cardiac cachexia^5^. The study showed that increased T1AM concentrations were independently associated with a reduced cardiac function, indicating a direct effect of this metabolite on the human heart. However, the pathophysiological significance of T1AM elevation, particularly in patients with cardiac cachexia, remains largely unknown.

T1AM has shown a substantial impact on the heart. Exogenous T1AM treatment was found to induce negative chronotropic and inotropic effects^6,7^. In contrast, the administration of T1AM during ischemia–reperfusion injury played a cardioprotective role by reducing the infarct size in the absence of any hemodynamic effect^8^. Overall, T1AM has been shown to exert opposite effects of its precursor thyroid hormones, including the induction of a hypometabolic state and notably hypothermic action^1,6^, which might be associated in some way with its cardiac effects. It would therefore be of great interest to investigate the direct impact of T1AM on cardiomyocytes per se (not merely whole heart tissue) and to identify the underlying molecular mechanisms.

We and others recently reported that low serum T3 levels correlate with high levels of B-type natriuretic peptide (BNP)^9–11^, a hormone produced in the heart that serves as a critical biomarker of the severity of heart failure^12^. Although various factors regulate BNP production and secretion from the myocardium in the setting of heart failure, increased T1AM as a result of accelerated T3 metabolism may also promote BNP production. In fact, T1AM treatment was found to reduce neurofibromatosis type 1 (Nf1) gene expression in cardiac cells^13^, and cardiac-specific knockout of Nf1 promotes cardiac hypertrophy along with natriuretic peptide (NP) production through the extracellular signal-regulated kinases (ERK) signaling pathway^14^, one of the three major mitogen-activated protein kinase (MAPK) cascades.

We recently detected the thermogenic actions of NP in a low-temperature-sensitive manner both in vitro^15^ and in vivo^16^, and even in a clinical setting^17^, while others have reported that the systemic administration of T1AM resulted in a substantial decrease in body temperature, namely hypothermic action^6^. To better understand the direct impact of T1AM on cardiomyocytes, we investigated whether or not T1AM treatment induces a change in intracellular temperature using a recently established fluorescent thermoprobe system^15,18–21^. We also explored the possibility of T1AM-induced BNP expression and elucidated the potential signaling pathway.

## Results

### The thermoprobe successfully detected the estimated intracellular temperature of neonatal rat cardiomyocytes (NRCM)

**Figure 1:**
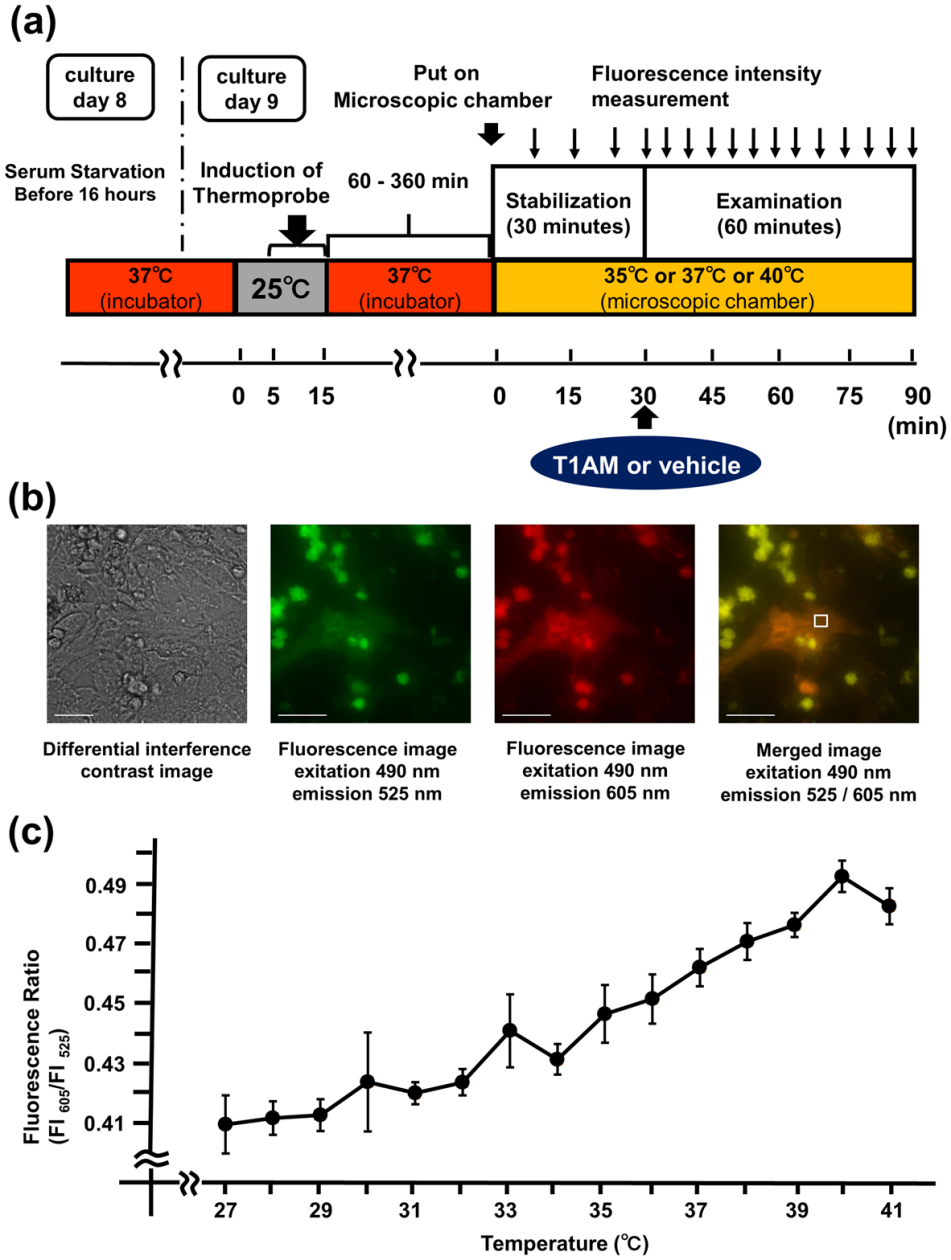
The estimated intracellular temperature measurement in NRCM using fluorescent polymeric thermometers. (**a**) The experimental protocol showing the duration and the time course of the fluorescent thermoprobe-detected temperature measurement. Neonatal rat cardiomyocytes (NRCM) treated with the cellular thermoprobe were incubated at 37 °C, and were placed in the microscopic chamber at 35 °C, 37 °C or 40 °C. (**b**) Representative microscopic images of NRCM treated with the fluorescent polymetric thermometer. A differential interference contrast image, a fluorescence image (490 nm excitation, 525 nm emission), a fluorescence image (490 nm excitation, 605 nm emission), and a merged image of fluorescence images with the sampling square of the measurement are also shown from left to right. Scale bar: 40 μm. (**c**) The calibration curve of the fluorescent polymetric thermometer in NRCM. The responses of the fluorescence ratio (605 nm/525 nm) were analyzed (n = 3).

The protocol of the present study is shown in Fig. 1a. Representative microscopic images of NRCM are shown in Fig. 1b. We confirmed that the fluorescent polymetric thermometers were successfully introduced into living NRCM in two different settings of excitation/emission. To measure the thermoprobe-detected intracellular temperature, cellular cytoplasm was selected in merged fluorescence image from two different values of excitation/emission (Fig. 1b)^15^. To confirm that the thermoprobe used in the current study could detect the subtle changes in the intracellular temperature, a calibration curve was prepared (Fig. 1c). The temperature-dependent change in the fluorescence intensity ratio of the cellular thermoprobe was observed at 605 to 525 nm (FI 605/FI 525), indicating that the fluorescence ratio measured by this polymetric thermometer was an appropriate marker for assessing the temperature in living cardiomyocytes^15^.

### T1AM decreased the thermoprobe-measured temperature in NRCM

**Figure 2:**
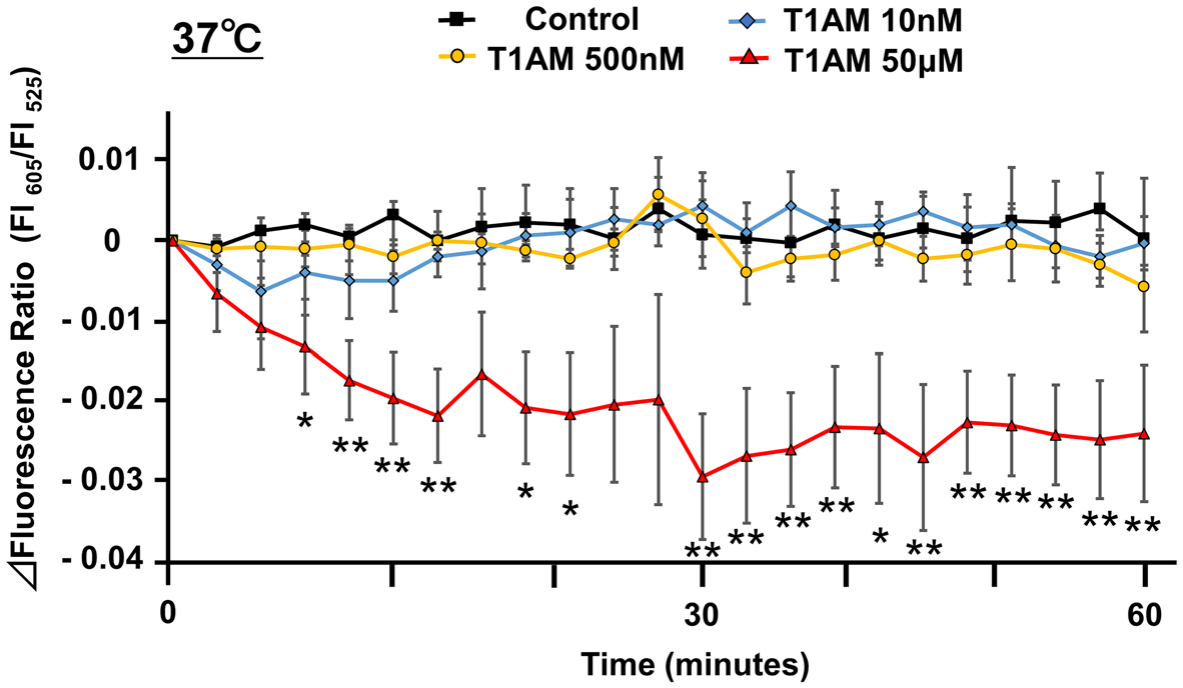
The profile of the thermoprobe-measured temperature change in NRCM incubated with T1AM at 37 °C. The thermoprobe-measured temperature was indicated by the Δfluorescence ratio (605 nm/525 nm). The changes of the fluorescence ratio after treatment with or without T1AM (10 nM, n = 5; 500 nM, n = 5; 50 μM, n = 5; Control, n = 6) were recorded every 2 min for the first 15 min and every 3 min for the remaining 45 min. *P < 0.05 and **P < 0.01 versus the controls at each time point.

The changes in the fluorescent thermoprobe-detected intracellular temperature in NRCM were measured for 60 min at 37 °C, and the time course of the temperature-dependent changes in the fluorescence ratio (indicated as Δfluorescence ratio) is shown in Fig. 2. Neither treatment with 10 or 500 nM (relatively physiological concentrations) of T1AM nor the equivalent solvent-treated control had a significant influence on the myocardial thermoprobe-measured temperature at 37 °C. In contrast, the fluorescence ratio showed a marked decrease with time following 50 μM of T1AM treatment (mean fluorescence ratio: 0.734 ± 0.041 at 0 min; 0.710 ± 0.044 at 60 min, *P* < 0.01). Accordingly, the ∆fluorescence ratios of the T1AM-treated (50 μM) groups were significantly lower than those of controls after 60 min of treatment (*P* < 0.01, Fig. 2). These data suggest the hypothermic effects of T1AM on cardiomyocytes. We also examined whether or not this hypothermic effect of T1AM was evident even at physiological concentrations in a high-temperature environment (Supplementary Fig. S1). However, neither 10 nor 50 nM of T1AM treatment significantly influenced the thermoprobe-measured temperature of NRCM, even at 40 °C, although 50 μM of T1AM consistently decreased the thermoprobe-measured temperature with time (mean fluorescence ratio: 0.763 ± 0.042 at 0 min; 0.735 ± 0.043 at 60 min, *P* < 0.01) (*P* < 0.01 vs. Control).

### Effects of T1AM on BNP expression in cardiomyocytes

**Figure 3:**
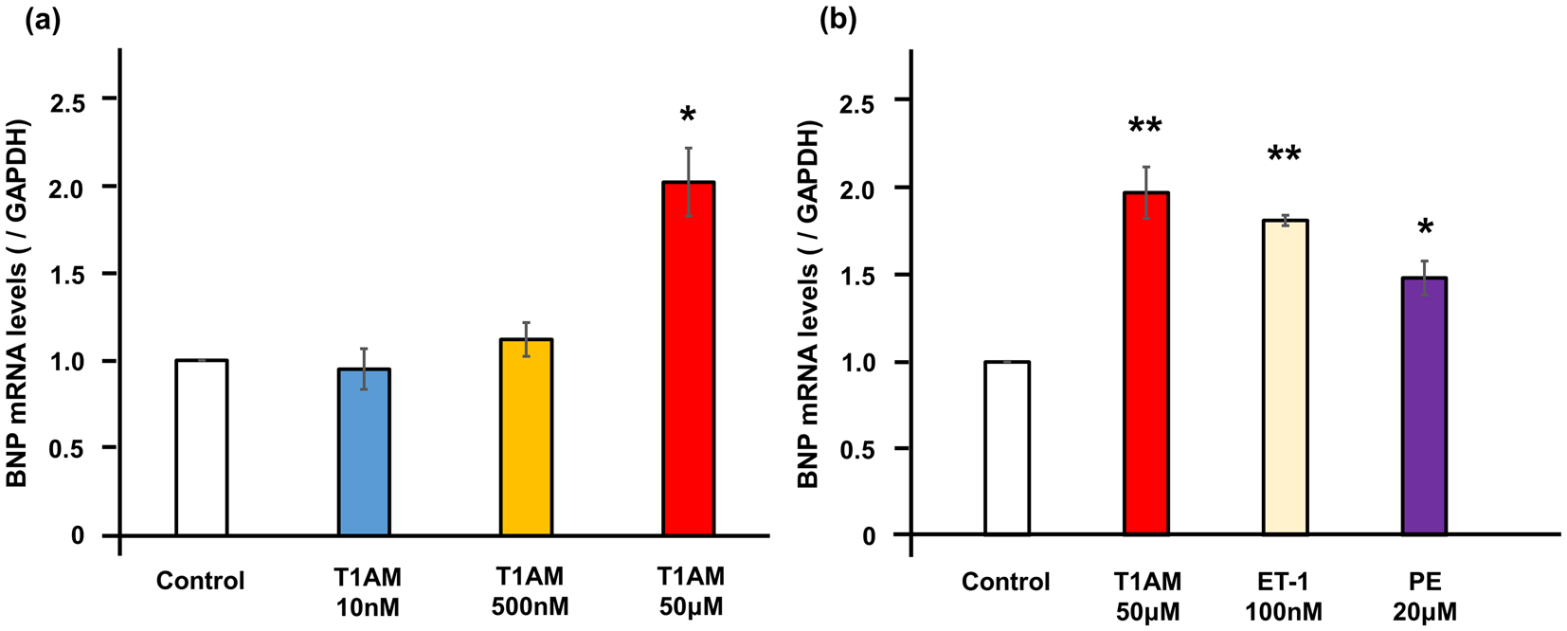
Effects of T1AM on BNP expressions in NRCM. (**a**) The quantification of the BNP gene expression levels in NRCM after 6 h of incubation with or without T1AM were shown (n = 5 each). (**b**) The quantification of the BNP gene expression levels in NRCM after six hours of incubation with T1AM (50 μM), endothelin-1 (100 nM), phenylephrine (20 μM), or vehicle were shown (n = 3 each). The qPCR data were normalized to GAPDH. The data are shown as the fold change normalized to the levels found in vehicle-treated cells (control). *P < 0.05 and **P < 0.01 versus control. ET-1, endothelin-1; PE, phenylephrine.

Given that serum T1AM levels were increased in patients with heart failure^5^, we next examined the effects of T1AM on BNP transcription in cardiomyocytes. After 6 h of treatment with T1AM (50 μM), the BNP mRNA levels were significantly increased compared to the equivalent solvent-treated control (1.95 ± 0.24-fold, *P* < 0.03, Fig. 3a), a finding not observed at lower concentrations of T1AM. The impact of T1AM on the BNP mRNA levels was comparable to that of endothelin-1 (ET-1) or phenylephrine (PE)—typical pro-hypertrophic hormones that increase BNP levels in cardiomyocytes^22,23^.

### Effects of NP and β-adrenergic receptor (β-AR) stimulants on thermoprobe-measured temperature in cardiomyocytes

We recently reported the thermogenic actions of NP as well as β-AR stimulants in a low-temperature-sensitive manner in brown adipocytes via the upregulation of intrinsic uncoupling protein (UCP)^15^. Given that T1AM increased the BNP expression levels in cardiomyocytes (Fig. 3), we investigated the effects of NP (A-type NP [ANP] was used as in our previous study^15^) and β-AR agonists (i.e., isoproterenol and CL316,243 [β_3_-AR agonist] were used as in our previous study^15^) on thermoprobe-measured temperature in NRCM under the same conditions as in our previous study^15^. In contrast to the thermogenic actions of ANP as well as β-AR stimulants in brown adipocytes^15^, neither of them significantly affected the thermoprobe-measured temperature in cardiomyocytes (Supplementary Fig. S2). Thus, cardiomyocytes and brown adipocytes may have a different mechanism of intracellular temperature regulation.

### T1AM regulates the thermoprobe-measured temperature and BNP expression through MEK-ERK pathway in cardiomyocytes

A previous study indicated that cAMP-PKA is a major downstream pathway of T1AM in pancreatic β-cells^24^, although another study failed to show the induction of cAMP by T1AM in adult rat hearts^7^. To elucidate the molecular mechanism underlying the findings noted above, we first investigated whether or not PKA signaling was involved. However, treatment with a PKA inhibitor did not influence the hypothermic effects of T1AM nor the T1AM-induced BNP expression in NRCM (Supplementary Figs. S3a and S3b). These data are in agreement with the findings shown in Supplementary Fig. S2 that β-AR agonists, which can activate cAMP-PKA signaling, did not significantly affect the thermoprobe-measured temperature in cardiomyocytes.

**Figure 4:**
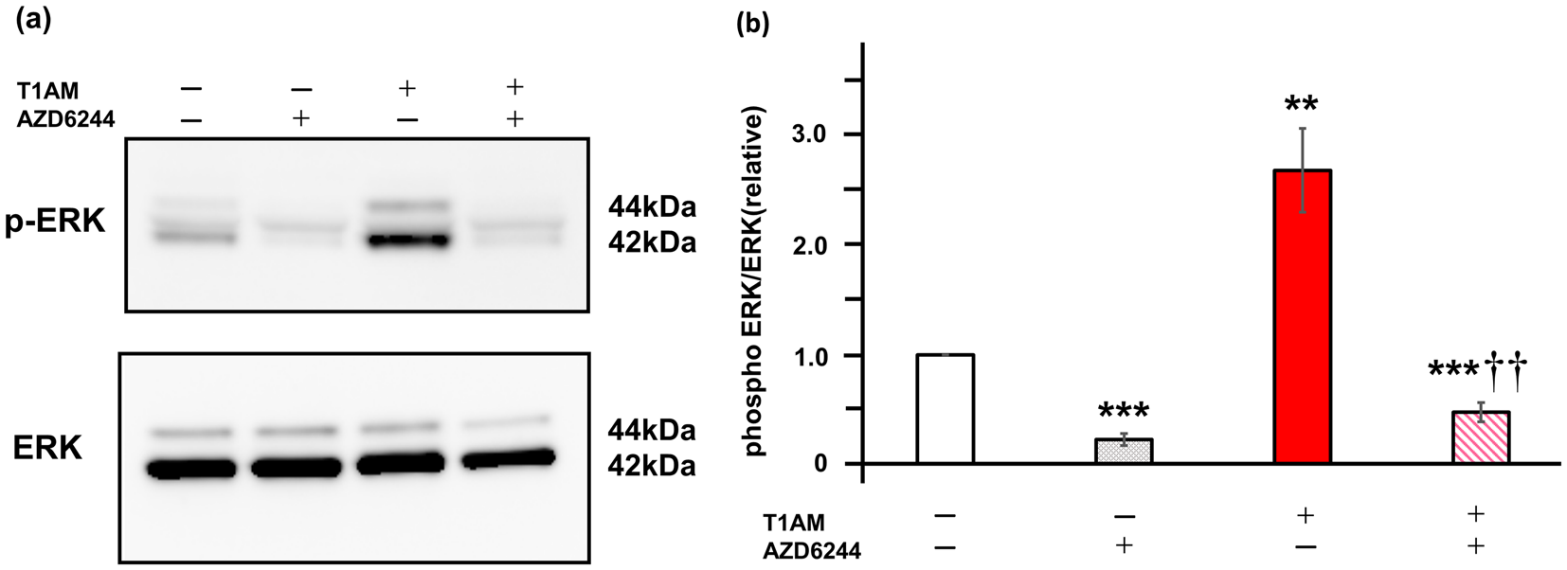
Effects of T1AM on ERK signaling in NRCM. (**a**) Phosphorylation of ERK was evaluated in NRCM treated with or without T1AM (50 μM) stimulated with either AZD6244 (MEK inhibitor, 500 nM) or vehicle for 6 h. Representative immunoblots obtained using the indicated antibodies are shown. (**b**) Averaged densitometry data normalized to the control at the same time points are shown in the bar graphs (n = 5 each). *P < 0.05, **P < 0.01 and ***P < 0.001 versus untreated control; ^††^*P* < 0.01 versus T1AM.

**Figure 5:**
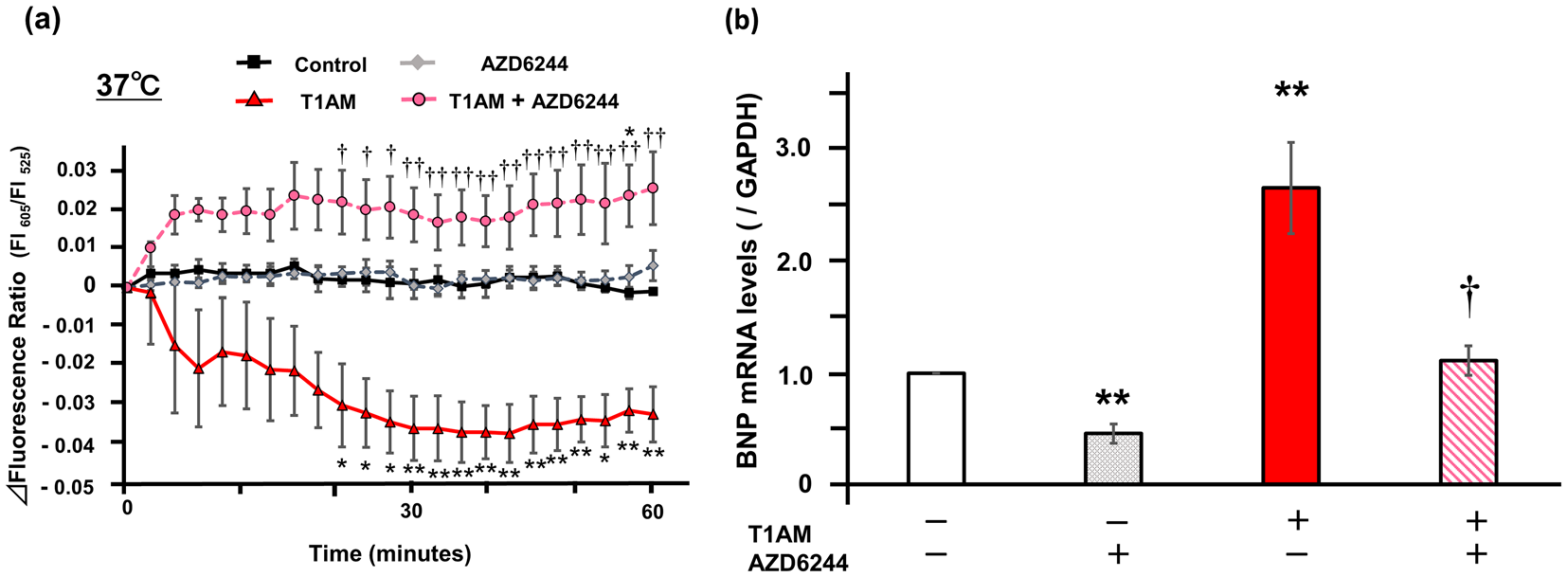
T1AM regulates thermoprobe-measured temperature and BNP expressions in NRCM through ERK signaling pathway. (**a**) The thermoprobe-measured temperature was indicated by the Δfluorescence ratio (605 nm/525 nm). The changes of the fluorescence ratio after treatment with or without T1AM (50 μM) stimulated with either AZD6244 (500 nM) or vehicle were recorded every 2 min for the first 15 min and every 3 min for the remaining 45 min at 37 °C (n = 3 each). *P < 0.05 and **P < 0.01 versus control; ^†^*P* < 0.05 and ^††^*P* < 0.01 versus T1AM at each time point. (**b**) The quantification of the BNP gene expression levels in NRCM after six hours of incubation with or without T1AM (50 μM) stimulated with either AZD6244 (500 nM) or vehicle were shown (n = 4 each). The qPCR data were normalized to GAPDH. The data are shown as the fold change normalized to the levels found in vehicle-treated cells (control). **P < 0.01 versus control; ^†^*P* < 0.05 versus T1AM.

We next investigated the role of MAPK kinase (MEK)/ERK pathway, another potential downstream cascade of T1AM in the heart, in the current findings^13,14^. We found that phosphorylation of ERK was significantly increased after T1AM treatment and substantially diminished by the inhibition of MEK1/2 with AZD6244 treatment (Fig. 4a,b). Accordingly, the decrease in thermoprobe-measured temperature that was induced by one-hour of T1AM treatment was cancelled by AZD6244 (Fig. 5a). Likewise, the T1AM-induced BNP expression was substantially suppressed by MEK1/2 inhibition (Fig. 5b). Although T1AM also increased phosphorylation of p38 and tended to increase phosphorylation of JNK, another two major MAPK signaling factors, the MEK inhibitor did not significantly influence these actions of T1AM (Supplementary Figs. S4a and S4b), suggesting that neither p38 nor JNK are involved in the T1AM-based regulation of intracellular temperature and BNP expression. These data indicate that T1AM decreases the thermoprobe-measured temperature and increases BNP mRNA levels in cardiomyocytes via the activation of the MEK/ERK pathway.

## Discussion

In the present study, we found that T1AM treatment significantly decreased the fluorescent thermoprobe-measured temperature but increased the BNP expression in cardiomyocytes. Intriguingly, T1AM treatment induced the marked phosphorylation of ERK, and inhibition of this ERK phosphorylation by a MEK inhibitor cancelled both the T1AM-induced thermoprobe-measured temperature decrease and BNP increase. The remarkable findings in the present study are that exogenous T1AM stimulus has a substantial direct impact on the regulation of the intracellular temperature as well as the NP expression in cardiomyocytes via activation of a common pathway of MEK/ERK.

A previous study showed that the administration of T1AM in vivo induces profound hypothermia^6^, although the precise mechanisms remain incompletely understood. Here, we found that T1AM decreases not only the in vivo systemic body temperature but also directly decreases the thermoprobe-measured temperature in cardiomyocytes. The direct effect of T1AM on the intracellular temperature regulation turned out to be mediated through the MEK/ERK pathway, but how the MEK/ERK pathway induces hypothermic action in cardiomyocytes remains unclear. These findings along with the observation that T1AM has negative inotropic and chronotropic effects on the heart^6,7^ suggest that this thyroid hormone metabolite promotes myocardial energy conservation, namely inducing a hibernating state for the heart. Furthermore, the current investigation uncovered new evidence that an endogenous compound can directly regulate myocardial intracellular temperature per se.

The BNP expression could be upregulated by a myocardial intracellular temperature decrease per se as a compensatory mechanism, given the thermogenic action of NP in a low-temperature-sensitive manner^15–17^. However, we did not observe an increase in BNP mRNA expression in cardiomyocytes after hypothermic incubation (the fold change of BNP mRNA levels normalized to the levels found in NRCM at 37 °C in the same time frame: [35 °C] 0.84-fold for 2 h, 0.85-fold for 6 h; [30 °C] 0.94-fold for 2 h, 0.85-fold for 6 h). Thus, the BNP upregulation observed in the current study might be due to the direct action of T1AM rather than its indirect effects via intracellular temperature decrease.

In contrast to the thermogenic actions of NPs as well as β-AR stimulants in brown adipocytes, as we reported previously^15^, neither ANP nor β-AR agonists significantly affected the thermoprobe-measured temperature in cardiomyocytes. The mechanisms underlying the intracellular temperature regulation of cardiomyocytes remain largely unknown. UCPs, particularly UCP1 (which is predominantly expressed in brown adipocytes) have roles in thermogenesis, although a previous study reported that T1AM did not significantly affect the UCP1 expression in brown adipose tissue^25^. Meanwhile, two isoforms (UCP2 and UCP3, but not UCP1) are located in the heart. Despite the high sequence similarity of UCP2 and UCP3 with UCP1, they are not implicated in adaptive thermogenesis and their physiological role is largely unknown^26^. Therefore, cardiomyocytes and brown adipocytes may have a different mechanism of intracellular temperature regulation. Likewise, the hypothermic action of T1AM is thought to be independent of the levels of NPs and their activity, at least in cardiomyocytes. It is possible that cell metabolism, such as glycolysis and mitochondrial respiration, has a close connection with thermogenesis. Thus, further studies are warranted to explore the impact of T1AM on myocardial energy metabolism using an extracellular flux analyzer and/or metabolome analyses.

Although T1AM showed a substantial impact on the heart, the molecular mechanism and its potential target are still largely unknown. T1AM was originally identified as a potent agonist of the G protein-coupled trace amine-associated receptor 1 (TAAR1)^6,24,27^. In pancreatic β-cells, the activation of TAAR1 by T1AM leads to cAMP generation, which then activates MEK/ERK signaling in an Epac and PKA-dependent manner^24^. TAAR1 also stimulates cAMP-dependent calcium flux, leading to CaMKII-dependent activation of MEK/ERK signaling. Given that augmented Ca^2+^-handling is associated with skeletal muscle thermogenesis^28^, it is possible that T1AM could also induce thermogenesis in the heart via enhanced Ca^2+^-handling by the activation of PKA or MAPK signaling. However, in the present study, we did not see any significant effects of PKA inhibitor on the actions of T1AM (Supplementary Figs. S3), which was consistent with a previous study^7^ showing that T1AM did not change the cAMP concentration in the heart, and that its negative cardiac inotropic and chronotropic effects were not modified by PKA inhibitor. In line with these studies, we found that β-AR agonists, which can activate cAMP-PKA signaling, leading to augmented Ca-handling, did not significantly affect the thermoprobe-measured temperature in cardiomyocytes (Supplementary Fig. S2). Taken together, these data suggest that cAMP-PKA signaling as well as Ca-handling per se have a limited impact on the T1AM-regulated thermoprobe-measured temperature and BNP expression in cardiomyocytes, at least in the present study.

In contrast, the present study showed that the MEK/ERK pathway is deeply involved in both the T1AM-induced decrease of thermoprobe-measured temperature and the upregulation of BNP in cardiomyocytes. Another previous in vitro study showed that T1AM reduced Nf1 gene expression in cardiac cells^13^, and cardiac-specific Nf1 knockout induced cardiac hypertrophy and NP production along with ERK signaling activation^14^. Thus, T1AM may activate MEK/ERK signaling by suppressing Nf1, which is a negative regulator of the MEK/ERK pathway. Given that ERK is known to have roles in mediating cardiomyocyte hypertrophy, it is possible that the administration of T1AM induces a hypertrophic response in cardiomyocytes. Therefore, it would be interesting to see whether T1AM treatment induces myocardial hypertrophy both in vitro and in vivo. Meanwhile, Li et al. recently reported that neurotensin acts to repress thermogenesis in brown adipocytes via ERK activation, thus suggesting that ERK signaling potentially has anti-thermogenic actions depending on cell type^29^. In any case, further studies are warranted to fully delineate the precise molecular mechanism by which T1AM activates MEK/ERK signaling as well as the mechanism by which ERK signaling induces the decrease in thermoprobe-measured temperature in cardiomyocytes. Likewise, the effects of T1AM on the thermoprobe-measured temperature were relatively rapid (presumably non-genomic action), while its effects on the BNP expression were mediated through genomic actions. Thus, the underlying mechanisms of the present two findings might differ despite sharing a common signaling pathway.

In the present study, a pharmacological dose rather than a physiological dose of T1AM showed a substantial impact. Recent studies have indicated that endogenous concentrations of T1AM lie in the nanomolar range^1–5,30–32^, whereas most studies have shown that T1AM exerts significant actions on the heart in the micromolar range^6–8,13^. Consistent with a series of previous studies, we noted no significant effects of T1AM in the nanomolar range, even under high-temperature conditions. However, the local cardiac tissue concentration of T1AM may be much higher, particularly under severe heart failure conditions, given that its subcellular distribution is unknown, so T1AM may be restricted to specific compartments^8,32^. At any rate, the dose used in the present study is comparable to that used in most previous studies showing significant effects on the myocardium^6–8,13^.

A major concern for single-cell thermometry, when performed inside living cells, is that the effects of various non-thermal factors on thermoprobes (e.g., pH, ionic strength, viscosity, as well as high-density macromolecular assemblies consisting of cytoskeletal networks, membranous organelles, RNAs, and also physical factors) cannot be ruled out^33^. Moreover, the possible alteration of myocardial energy metabolism in response to T1AM might have some influence on the detection sensitivity of the thermoprobe. Thus, the thermoprobe-measured temperature in the present study indicates the “estimated” intracellular temperature. Although the fluorescent polymeric thermometer used in the present study was recently developed^18–20^ and has recently become commercially available^15,21^, it would be better to further evaluate whether the observed changes actually indicate intracellular temperature changes using other probes and/or modalities^20,34,35^.

In summary, T1AM decreases the fluorescent thermoprobe-measured temperature and increases the BNP expression in cardiomyocytes through the activation of the MEK/ERK pathway. The biosynthetic pathway of T1AM as well as its biological effects (including precise molecular signaling pathway targets) are still not fully understood; however, the present study offers new insight into its direct actions on cardiomyocytes. Furthermore, although the pathophysiological implications of the elevation of serum T1AM levels in heart failure subjects with cachexia remain unclear, the present findings suggest that the hypothermic actions of T1AM may contribute to reduced energy demand and preservation of energy metabolism as a compensatory mechanism for adaptive regulation under severe cardiac conditions.

## Methods

### Primary cultures of isolated NRCM

All animal procedures conformed to the National Institutes of Health Guide for the Care and Use of Laboratory Animals and were approved by the Animal Research Committee at the Jikei University School of Medicine (2021-023). All animal experiments were carried out in accordance with the ARRIVE guidelines. Primary myocyte cultures were prepared from 1-day-old Wister rat heart ventricles using Neonatal Cardiomyocyte Isolation System (Worthington Biochemical Corp), plated in 60 mm or 35 mm collagen-coated dishes as previously described^36,37^. Cardiomyocytes were cultured in Dulbecco’s modified eagle medium (DMEM) (Gibco) containing 10% horse serum, 5% fetal bovine serum, 1% penicillin–streptomycin, and 200 μM bromodeoxyuridine at 37 °C in humidified air with 5% CO_2_. The medium was then changed to serum-free DMEM, and the cells were incubated for 16 h before all experiments.

### The measurement of the intracellular temperature using fluorescent polymeric thermometers

The estimated intracellular temperature in NRCM was measured using Cellular Thermoprobe for Fluorescence Ratio (Funakoshi Corp.) according to the manufacture’s protocol as previously described in more detail^15^. NRCM prepared on a collagen-coated 35 mm dish, as described above. A Delta Vision (Airix Co.) fluorescence microscope was used to detect the thermoprobe in order to determine the fluorescence ratio. When measuring the fluorescence intensity, each dish was set up on the microscopic chamber at 35 °C, 37 °C or 40 °C with 5% CO2. Where indicated, 500 nM of AZD6244 (Cayman Chemical), MEK inhibitor, or 10 μM of H-89 (Cayman Chemical), PKA inhibitor, was added to the medium 30 min prior to the stabilization period (Fig. 1a). T1AM (Sigma-Aldrich) was dissolved in 60% dimethylsulfoxide (DMSO)/40% saline, and AZD6244 and H-89 were dissolved in DMSO. The solvent concentration was identically maintained in the control group. The cellular cytoplasm in which the thermoprobe was substantially introduced was selected and fixed under the microscope using a sampling square (Fig. 1b). The fluorescence intensity (FI) was measured every 6 min during stabilization period with excitation at 490 nm and dual emission at 525 nm and 605 nm, and the fluorescence ratio was calculated as FI 605 nm divided by FI 525 nm. After stabilization for 30 min, either T1AM (10 nM, 50 nM, 500 nM, or 50 μM) or the equivalent amount of DMSO/saline was added directly to the indicated samples. The FI was subsequently measured every 2 min for the first 15 min and every 3 min for the remaining 45 min. Where indicated, either 100 nM of ANP (carperitide, provided by Daiichi-Sankyo Pharmaceutical Co.), 100 nM of isoproterenol (Sigma-Aldrich), or 500 nM of CL316,243 (Tocris, Bristol, UK) (those were dissolved in distilled water, and the equivalent amount of distilled water was added to the control) was added directly to the indicated samples. The FI was subsequently measured every 6 min for 60 min^15^. To evaluate the time-dependent change of the thermoprobe-measured temperature, the Δfluorescence ratio was calculated for each sample as the fluorescence ratio at the indicated time minus the ratio at 0 min^15^.

### The determination of the calibration curve

After cell pellets of NRCM were collected from 35 mm dishes, the cell extract with the thermoprobe was loaded in a 96-well plate and the FI was measured by a plate reader equipped with temperature control (EnSpire, Perkin Elmer), as previously described in more detail^15^. The fluorescence ratio (excitation at 490 nm, emission ratio at FI 605 nm/FI 525 nm) was measured after the temperature of the plate chamber became steady (27–41 °C). The fluorescence ratio against the temperature was plotted to obtain the calibration curve^15^.

### RNA isolation, reverse transcription and real-time polymerase chain reaction (PCR)

After 6 h of stimulation by the indicated hormone (10 nM, 500 nM or 50 μM of T1AM, 100 nM of ET-1, and 20 μM of PE), each dish was snap frozen. Where indicated, NRCM were first pretreated with 500 nM of AZD6244 (Cayman Chemical) or 10 μM of H-89 30 min prior to the indicated hormone stimulation. Total RNA was extracted from the frozen cells using TRIzol reagent (Invitrogen) and a quantitative real-time PCR was performed using a StepOnePlus Real-time PCR System and the StepOne Software program (Applied Biosystems), as described previously^15,37^. The real-time PCR protocol consisted of one cycle at 95 °C for 20 s followed by 40 cycles at 95 °C for 1 s and 60 °C for 20 s using the primers for NPPB (Applied Biosystems, Rn00580641_m1) and GAPDH (Applied Biosystems, Rn01775763_g1). The transcriptional levels were determined using the ΔΔCt method with normalization to GAPDH^15^.

### Immunoblotting

After 6 h of T1AM stimulation, each dish was snap frozen. Immunoblotting was performed as previously described^15,36^ with rabbit monoclonal anti phospho-ERK (1:2000, Cell Signaling Technology, #4370), anti ERK (1:1000, Cell Signaling Technology, #4695), anti phospho-p38 (1:1000, Cell Signaling Technology, #4511), anti p38 (1:1000, Cell Signaling Technology, #8690), anti phospho-JNK (1:1000, Cell Signaling Technology, #4668), or anti JNK (1:1000, Cell Signaling Technology, #9252). The signals were detected using chemiluminescence.

### Statistical analyses

The data are presented as the mean ± standard error of the mean (SEM) of the indicated number of independent experiments. The statistical analyses included a one-way ANOVA followed by Bonferroni’s test for the correction of multiple comparisons; Student’s t-test was used for the comparison of two sets of data. A value of *P* < 0.05 was considered to be significant.

## Supplementary Information

Supplementary Information.

## Data Availability

The datasets generated during and/or analyzed during the current study are available from the corresponding author on reasonable request.
