# Supplementary material for: Substantial impact of 3-iodothyronamine (T1AM) on the regulations of fluorescent thermoprobe-measured cellular temperature and natriuretic peptide expression in cardiomyocytes
Source: Sci Rep. 2022 Jul 26;12:12740. doi: 10.1038/s41598-022-17086-2 (PMC9325765; doi:10.1038/s41598-022-17086-2)
Supplement: Supplementary file 1 — Supplementary Information. [file 41598_2022_17086_MOESM1_ESM.docx]

**Supplementary Information**

**Substantial impact of 3-iodothyronamine (T1AM) on the regulations of fluorescent thermoprobe-measured cellular temperature and natriuretic peptide expression in cardiomyocytes.**

Hirotake Takahashi^1^, Tomohisa Nagoshi*^1^, Haruka Kimura^1^, Yoshiro Tanaka^1^, Rei Yasutake^1^, Yuhei Oi^1^, Akira Yoshii^1^, Toshikazu D. Tanaka^1^, Yusuke Kashiwagi^1^, and Michihiro Yoshimura^1^

^1^ Division of Cardiology, Department of Internal Medicine,

The Jikei University School of Medicine

***Correspondence to:** Tomohisa Nagoshi, M.D.,Ph.D.

E-mail: [tnagoshi@jikei.ac.jp](mailto:tnagoshi@jikei.ac.jp)


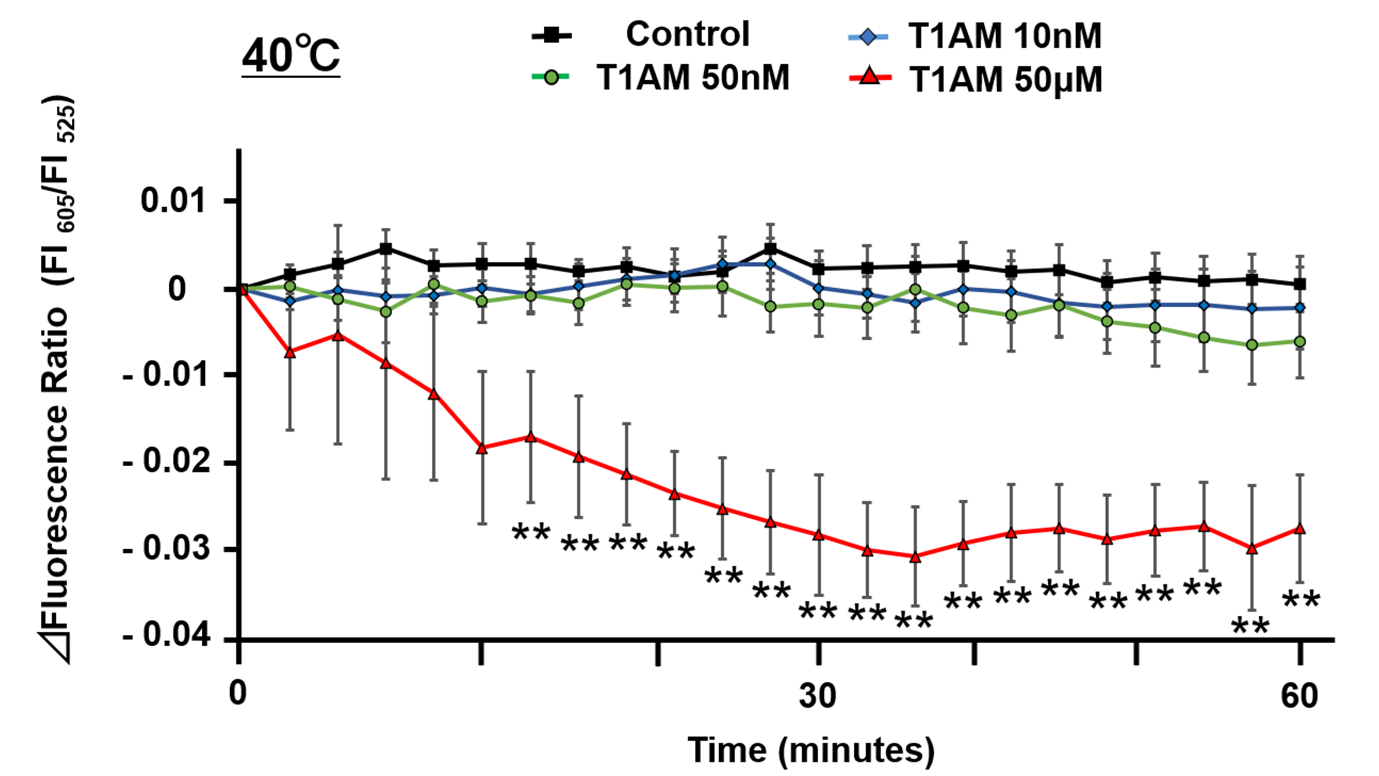


**Fig. S1 The profile of the thermoprobe-measured temperature change in neonatal rat cardiomyocytes (NRCM) incubated with T1AM at 40**°**C.** The thermoprobe-measured temperature was indicated by the Δfluorescence ratio (605 nm/525 nm). The changes of the fluorescence ratio after treatment with or without T1AM (10 nM, n=7; 50 nM, n=7; 50 μM, n=4; Control, n=9) were recorded every 2 minutes for the first 15 minutes and every 3 minutes for the remaining 45 minutes. *P < 0.05 and **P < 0.01 versus the controls at each time point.


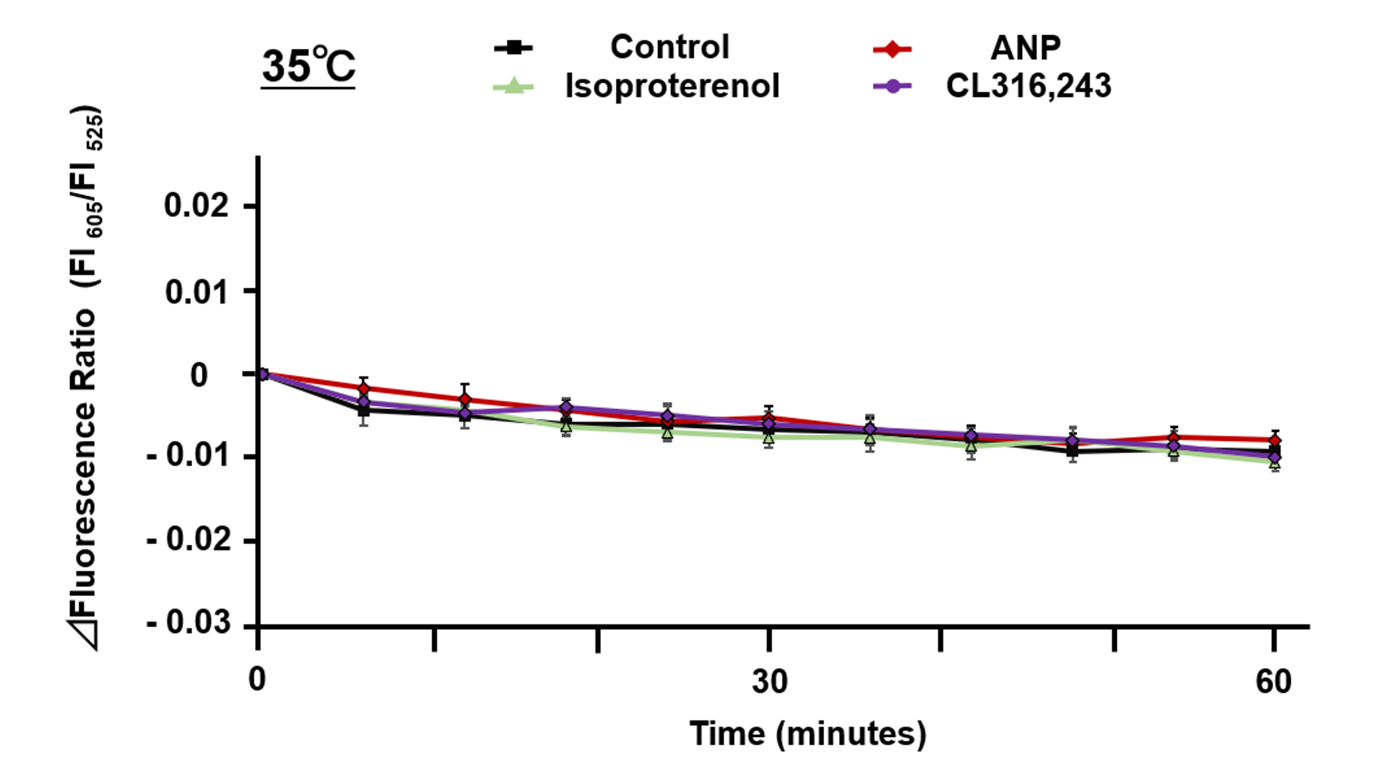


**Fig. S2 The profile of the thermoprobe-measured temperature change in NRCM incubated with ANP, isoproterenol, or CL316,243 at 35** °**C.** The thermoprobe-measured temperature was indicated by the Δfluorescence ratio (605 nm/525 nm). The changes of the fluorescence ratio after treatment with ANP (100 nM), Isoproteronol (100 nM), or CL316,243 (500 nM) were recorded every 6 minutes (n=3 each). *P < 0.05 and **P < 0.01 versus the controls at each time point.

**
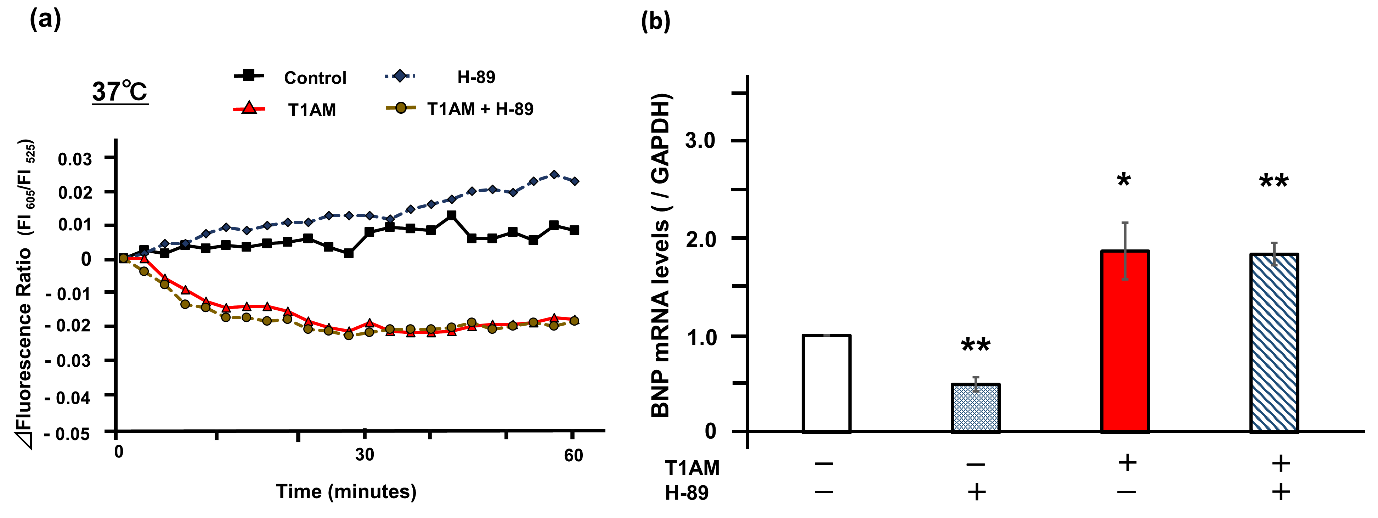
**

**Fig. S3 PKA signaling is not involved in T1AM-regulated thermoprobe-measured temperature and BNP expressions in NRCM. (a)** The thermoprobe-measured temperature was indicated by the Δfluorescence ratio (605 nm/525 nm). The changes of the fluorescence ratio after treatment with or without T1AM (50 μM) stimulated with either H-89 (PKA-inhibitor, 10 μM) or vehicle were recorded every 2 minutes for the first 15 minutes and every 3 minutes for the remaining 45 minutes at 37°C (n=2 each). **(b)** The quantification of the BNP gene expression levels in NRCM after six hours of incubation with or without T1AM (50 μM) stimulated with either H-89 (10 μM) or vehicle were shown (n=3 each). The qPCR data were normalized to GAPDH. The data are shown as the fold change normalized to the levels found in vehicle-treated cells (control). *P < 0.05 and **P <0.01 versus control.


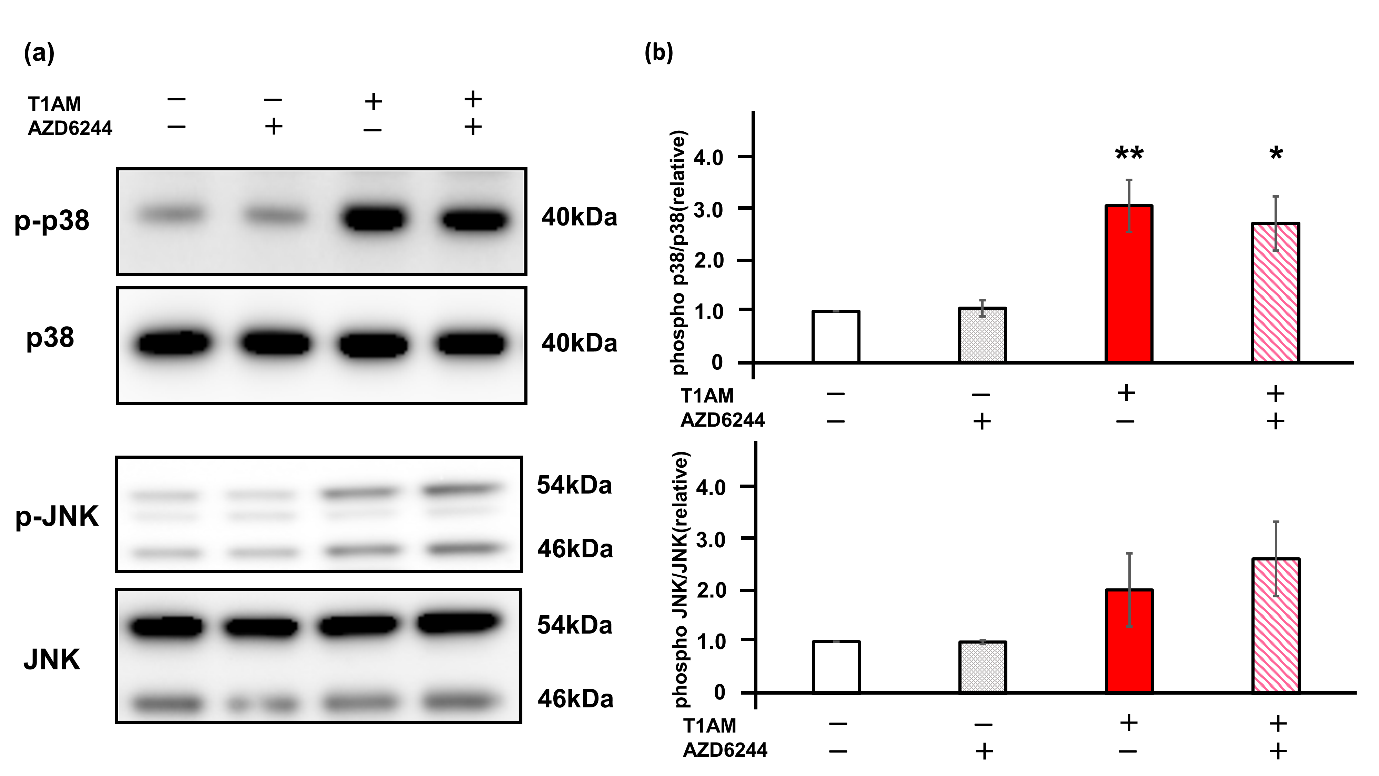


**Fig. S4 Effects of T1AM on p38 and JNK signalings in NRCM. (a)** Phosphorylations of p38 and JNK were evaluated in NRCM treated with or without T1AM (50 μM) stimulated with either AZD6244 (MEK-inhibitor, 500 nM) or vehicle for 6 hours. Representative immunoblots obtained using the indicated antibodies are shown. **(b)** Averaged densitometry data normalized to the controls at the same time points are shown in the bar graphs (n=5 each). *P < 0.05, **P <0.01 versus untreated control.


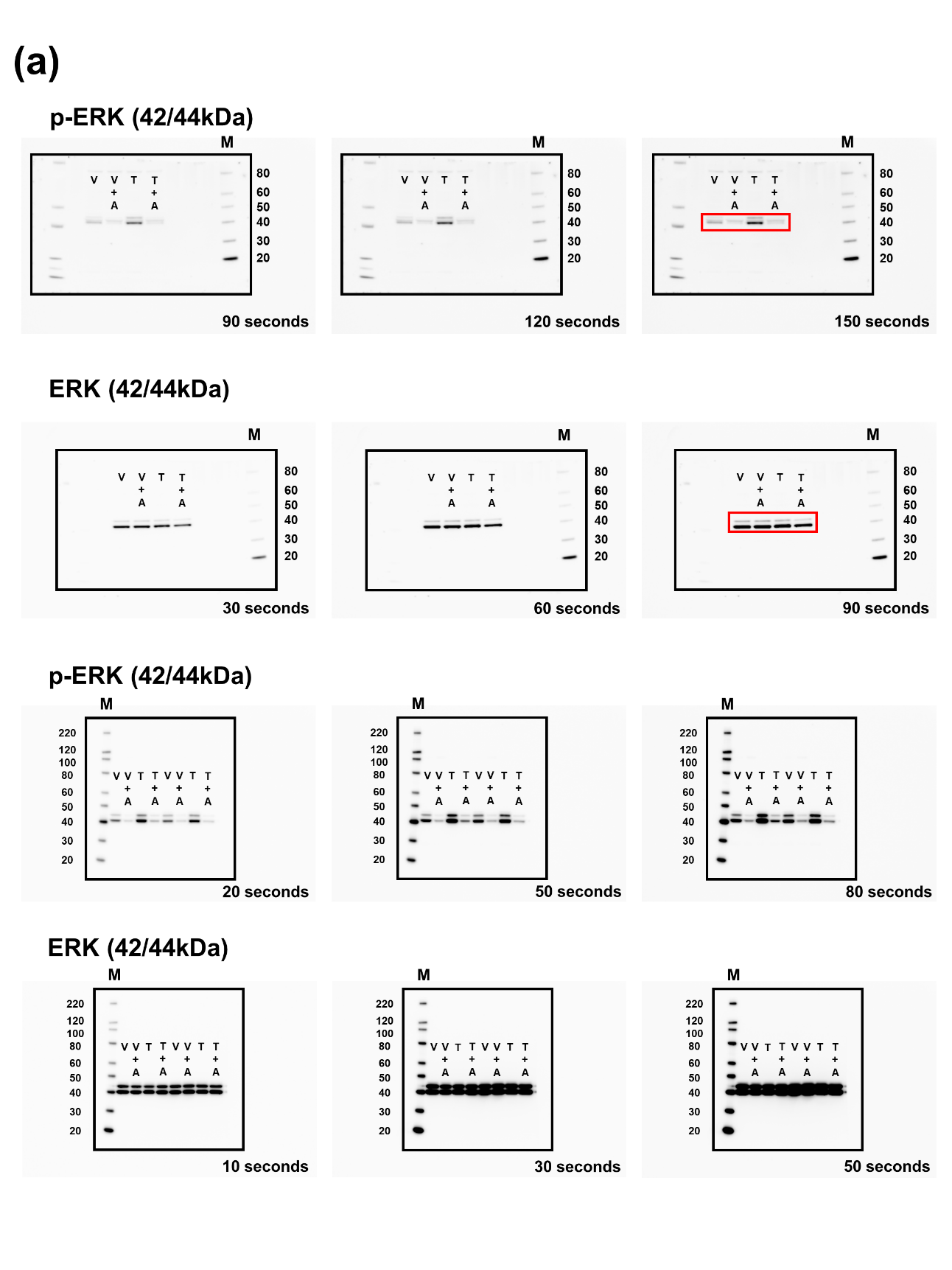


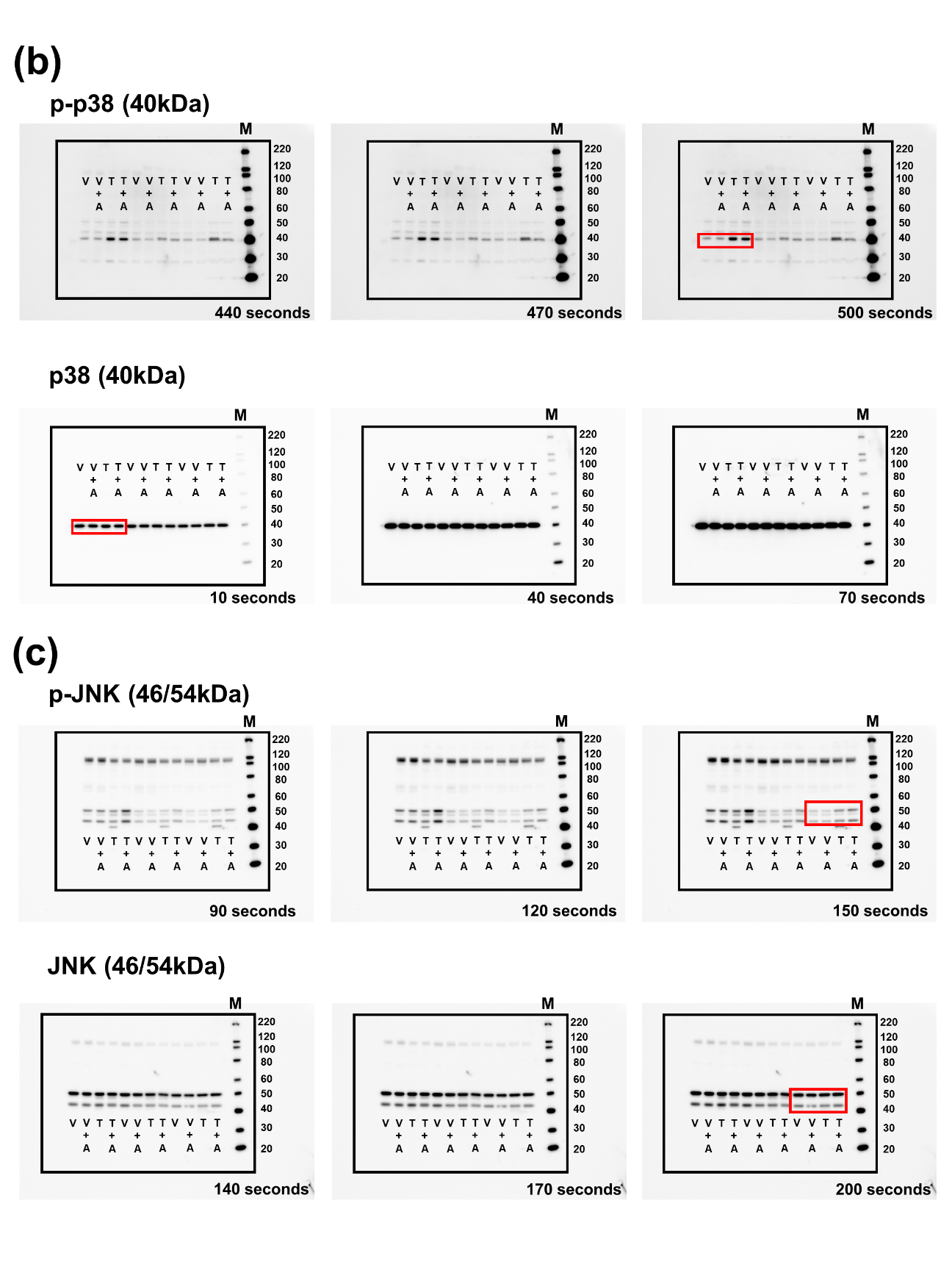


**Fig. S5 Full-length gels of immunoblottings.**

Western blotting of the indicated antibodies. Black boxes correspond to the edges of original blots. Red boxes correspond to the cropped samples shown in Fig. 4 and Suppl. Fig. S3. The number of seconds indicate exposure time. V, Vehicle; T; T1AM; A, AZD6244; kDa, kilo Dalton; M, MagicMark XP Western Protein standard, a molecular weight marker.
